# Hypermethylation of MEG3 promoter correlates with inactivation of MEG3 and poor prognosis in patients with retinoblastoma

**DOI:** 10.1186/s12967-017-1372-8

**Published:** 2017-12-29

**Authors:** Yali Gao, Peng Huang, Jun Zhang

**Affiliations:** 1 Department of Ophthalmology, The Second Clinical Medical College (Shenzhen People’s Hospital), Jinan University, Shenzhen, 518020 People’s Republic of China; 2 Department of Obstetrics and Gynecology, The Second Clinical Medical College (Shenzhen People’s Hospital), Jinan University, Shenzhen, 518020 People’s Republic of China

**Keywords:** Retinoblastoma, Long non-coding RNA, MEG3, Epigenetic, DNA methylation, Prognosis

## Abstract

**Background:**

In our previous study, we revealed that MEG3 was a tumor suppressor gene in retinoblastoma and inhibited proliferation of retinoblastoma cells by regulating the activity of the Wnt/β-catenin pathway. Here, we further explored the mechanism of MEG3 inactivation in retinoblastoma.

**Methods:**

MSP and qRT-PCR were performed to detect the methylation status of MEG3 promoter and levels of MEG3 expression, respective. To further explore relationship between MEG3 expression and epigenetic modifications, 5-Aza-CdR was used to interfere with DNA methylation. In addition, we evaluated proliferation, apoptosis and the expression of β-catenin via CCK-8, flow cytometric analysis and western blot analysis, respective.

**Results:**

Hypermethylation of MEG3 promoter was observed more frequently in retinoblastoma tissues and was highly associated with low MEG3 expression and poor survival of retinoblastoma patients. We also provided evidence demonstrating that hypermethylation of MEG3 promoter depressed MEG3 expression, promoted proliferation, inhibited apoptosis and increased β-catenin expression of retinoblastoma cells in vitro.

**Conclusions:**

Our present study indicates that promoter silencing by hypermethylation may account for the loss of MEG3 expression and predict poor prognosis.

## Background

Retinoblastoma is a most common intraocular malignant neoplasm that originates from the developing retina in children < 5 years of age with incidence of 1 in 14,000–20,000 live births [1]. Despite extensive research efforts, retinoblastoma associated mortality rate remains around 70% due to delayed detection and lack of effective therapies, especially in developing country [2]. These factors underscore the importance of correlating disease biology with patient prognosis and therapeutic regimens.

Long non-coding RNAs (lncRNAs) are transcripts which longer than 200 nt, do not encode proteins and play a crucial role in regulating gene expression at various levels [3, 4]. Maternally expressed gene 3 (MEG3), which located in chromosome 14q32 and encodes a lncRNA, represents as an important tumor suppressor gene in human [5, 6]. Although MEG3 is expressed in many normal tissues, it is lost in an expanding list of human cancers such as non-small cell lung cancer, cervical cancer and bladder cancer [7–9]. More important, our previous study has proved that down-regulation of MEG3 contributes to retinoblastoma progression by promoting the activity of Wnt/β-catenin pathway [10]. However, the exact mechanics of MEG3 lost in retinoblastoma remain elusive.

To identify mechanics of MEG3 lost, we first examined the methylation level of MEG3 promoter in 63 retinoblastoma tissues by MSP and evaluated the relationship between MEG3 methylation and prognosis of retinoblastoma patients. Furthermore, we assessed the effect of MEG3 methylation on MEG3 expression, cell growth and Wnt/β-catenin pathway. These results thus aid in understanding the role of MEG3 methylation in retinoblastoma progression and may provide clues to new therapeutic strategies.

## Methods

### Clinical samples

Sixty-three patients with retinoblastoma recruited in this study were the same as our previous study [10]. The collection of human tissue samples was approved and supervised by the Ethics Committee of our Hospital and informed consent was obtained from each patient.

### Cell culture

The human retinoblastoma cell lines Weri-Rb1 and Y79 were purchased from the Shanghai Institute of Biochemistry and Cell Biology of the Chinese Academy of Sciences (Shanghai, China) and cultured with RPMI 1640 medium (Gibco, Gaithersburg, MD, USA) supplemented with 10% fetal bovine serum (Gibco) at 37 °C in 5% CO_2_.

### Transfection of pcDNA3.1 and siRNA

PcDNA-MEG3 and si-MEG3 were synthesized as previously described [10]. The cells were transiently transfected with pcDNA-MEG3, si-MEG3 and negative control respectively using Lipofectamine 2000 (Invitrogen, California, USA) according to the manufacturer’s instructions. Transfected cell lines were harvested 48 or 72 h post-transfection for following assays.

### Quantitative real-time PCR

Total RNA was purified from tissues and cells using the Trizol reagent (TaKaRa, Otsu, Japan) and then was used to synthesize cDNA by PrimeScriptTM RT reagent Kit (TaKaRa, Otsu, Japan) according to the manufacturer’s protocol respectively. Quantitative real-time PCR (qRT-PCR) was performed using SYBR Premix Ex Taq II Kit (TaKaRa, Otsu, Japan) on CFX96 Real-Time PCR Detection System (Bio-Rad, Hercules, California, US) following the manufacturer’s protocol. The geometric means of GAPDH and β-Actin were applied as a separate normalizer and the fold change for the MEG3 expression level was calculated using 2^−ΔΔCt^ method. The PCR primer for MEG3 was as follows: 5′-CCTTCCATGCTGAGCTGCT-3′ (forward) and 5′-TGTTGGTGGGATCCAGGAAA-3′ (reverse).

### Western blot analysis

Western blot analysis was performed following our previous descriptions [10]. Briefly, 50 μg total protein lysates extracted from cells were separated with 10% SDS-PAGE gel electrophoresis and transferred to a PVDF membrane (Roche, Basel, Switzerland). First, the membrane was probed with anti-β-catenin and anti-β-actin antibody (1:1000, Cell Signaling Technology). Then the membrane was probed with HRP Goat-anti-Rabbit antibody (1:2000; Santa Cruz Biotechnology, Dallas, TX, USA) and detected by enhanced chemiluminescence reagent (ECL, Thermo Scientific) according to the manufacturer’s instructions. The β-Actin was used as a protein-loading control.

### Cell proliferation assay and flow cytometric analysis

Cell proliferation was assessed using a CCK-8 assay kit (Dojindo, Kumamoto, Japan) and flow cytometric analysis was performed using an Annexin V-FITC Apoptosis Detection Kit (eBioscience, San Diego, CA, USA) as described previously [10]. In CCK-8 assay, the plate was read using a multifunctional microplate reader SpectraMax M5 (Molecular Devise, CA, USA) at a wavelength of 450 nm at 0, 24, 48, and 72 h time points. In flow cytometric analysis, the ratio of early apoptotic cells was calculated by Cell Quest Pro software (BD Bioscience, CA, USA).

### Treatment of Weri-Rb1 and Y79 cells with 5-Aza-2-deoxycytidine

Weri-Rb1 and Y79 cells were seeded onto 6-well plates at 2.5 × 10^5^ cells per well and cultured in RPMI 1640 containing 0, 2.5, 5 or 10 μM 5-aza-2-deoxycytidine (5-Aza-CdR, Sigma-Aldrich, USA) for 5 days as our previously described [10]. The cells treated with 5-Aza-CdR were harvested and used for detection of MEG3 expression and functional assays.

### Methylation specific PCR

Genomic DNA was extracted from tissues and cells using a QIAmp DNA Mini kit (Qiagen, German) according to the manufacturer’s directions. For converting unmethylated cytosines to uracils, the extracted DNA was treated with bisulfite prior to methylation specific PCR (MSP) using an EpiTect Bisulfite kit (Qiagen) in accordance with the manufacturer’s instructions. Then the MSP amplifications were performed using an EpiTect MSP kit (Qiagen) at following conditions: 95 °C 15 min; 94 °C 30 s, 70 °C 30 s,72 °C 30 s, 5 cycles; 94 °C 30 s, 65 °C 30 s, 72 °C 30 s, 5 cycles; 94 °C 30 s, 60 °C 30 s, 72 °C 30 s, 30 cycles; 72 °C 7 min. Finally, the PCR products were electrophoresed on a 2.5% agarose gel, stained with ethidium bromide and visualized under ultraviolet illumination (ChemiDoc XRS, Bio-Rad, CA, USA). The sense and antisense primers for the methylated promoter of MEG3 (M) were 5′-GTT AGT AAT CGG GTT TGT CGG C and 5′-AAT CAT AAC TCC GAA CAC CCG CG, respectively. The primers for unmethylated promoter of MEG3 (U) were 5′-GAG GAT GGT TAG TTA TTG GGG T (sense) and 5′-CCA CCA TAA CCA ACA CCC TAT AAT CAC A (antisense).

### Statistical analysis

All experiments were performed in triplicate and data was presented as mean ± SEM. Statistical analyses were performed using SPSS software 11.0 (SPSS, Chicago, IL, USA) and GraphPad Prism 5.0 (GraphPad Software, San Diego, CA, USA). The difference among the groups in these assays was estimated by Student’s t test or LSD test. The associations between MEG3 methylation and retinoblastoma were evaluated by Chi square test. P value of < 0.05 was considered to indicate statistically significant.

## Results

### Methylation levels of MEG3 promoter in retinoblastoma tissues

**Fig. 1:**
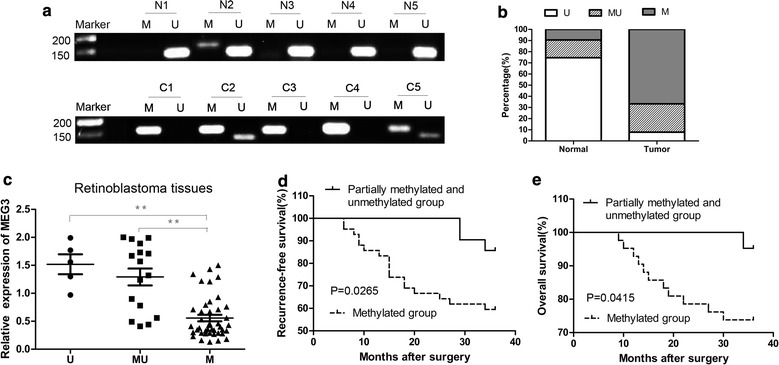
The level and clinical significance of MEG3 methylation in retinoblastoma. **a** The methylated (160 bp) and unmethylated (120 bp) PCR products were designated as M or U, respectively. Retinoblastoma tissues C1–C5 and corresponding non-tumor tissues N1–N5 was used as examples. **b** The frequency of methylation of MEG3 promoter in retinoblastoma tissues and corresponding non-tumor tissues was shown. **c** Correlation of promoter methylation and MEG3 expression in retinoblastoma. **d** and **e** Kaplan–Meier plots for patient’s survival according to the methylation status of MEG3 promoter. The log-rank test was used to calculate the P value. **P < 0.01

In the current study, the methylation status of MEG3 promoter in 63 retinoblastoma tissues and corresponding non-tumor tissues was detected by MSP. Methylated pattern (M), partially methylated pattern (M and U) and unmethylated pattern (U) was detected in 42 (66.7%), 16 (25.4%) and 5 (7.9%) cases of retinoblastoma, respectively (Fig. 1a, b). By comparison, 6 (9.5%) cases with methylated pattern, 10 (15.9%) cases with partially methylated pattern and 47 (74.6%) cases with unmethylated pattern were observed in corresponding non-tumor tissues (Fig. 1a, b). The difference between retinoblastoma tissues and corresponding non-tumor tissues was statistically significant (Table 1, P < 0.001). Moreover, the patients were divided into methylated group and partially methylated and unmethylated group according to methylation levels. A significant lower expression of MEG3 was detected in methylated group compared with the partially methylated and unmethylated group (Fig. 1c, P < 0.01).

**Table 1:** MEG3 methylation and retinoblastoma

Tissues	Methylation status		χ^2^	P	R	OR (95% CI)
	M	MU, U				
Retinoblastoma	42	21	43.615	< 0.001	0.588	19.000 (7.053–51.183)
Normal	6	57				

*OR* odds ratio

### The clinical significance of MEG3 methylation

As shown in Table 1, our results indicated that hypermethylation of MEG3 promoter was significantly associated with retinoblastoma (Table 1, R = 0.588). Moreover, the date also revealed that hypermethylation of MEG3 promoter was a risk factor for retinoblastoma (Table 1, OR = 19). Furthermore, examination of the survival by Kaplan–Meier curves according to the methylation status of MEG3 promoter revealed that the patients with methylated MEG3 had poorer recurrence free and overall survival than the patients with partially methylated and unmethylated MEG3 (Fig. 1d, e). More important, Univariate analysis for recurrence free survival revealed that MEG3 methylation was a prognostic factor (Table 2). Then multivariate analysis confirmed that MEG3 methylation was retained as an independent prognostic indicator for patients with GC in addition to the presence of International Intraocular Retinoblastoma Classification (IIRC) stages, nodal or distant metastasis and optic nerve invasion (Table 2).

**Table 2:** Univariate and multivariate cox regression analyses for recurrence free survival

Risk factors	Univariate analysis			Multivariate analysis		
	HR	P value	95% CI	HR	P value	95% CI
MEG3 methylation	3.624	0.040	1.060–12.389	4.639	0.021	1.260–17.087
IIRC stage (A/B/C/D, E)	17.901	< 0.001	5.195–61.678	7.579	0.018	1.418–40.511
Nodal or distant metastasis (negative, positive)	12.174	< 0.001	4.725–31.369	4.247	0.008	1.466–12.303
Optic nerve invasion (negative, positive)	13.988	< 0.001	4.072–48.045	2.406	0.314	0.436–13.279

*HR* hazard ratio

### MEG3 expression is modulated by promoter methylation

**Fig. 2:**
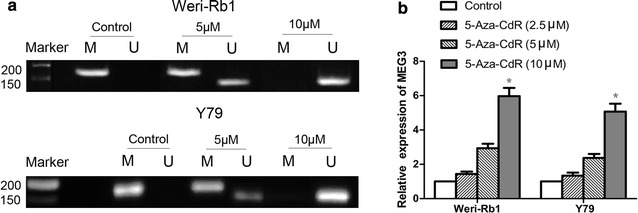
The effect of 5-Aza-CdR on MEG3 methylation and MEG3 expression in Weri-Rb1 and Y79 cells. **a** Hypermethylation of MEG3 promoter was observed in Weri-Rb1 and Y79 cells. 5-Aza-CdR decreased the methylation level of MEG3 promoter. **b** 5-Aza-CdR down-regulated the expression level of MEG3. *P < 0.05, **P < 0.01

**Fig. 3:**
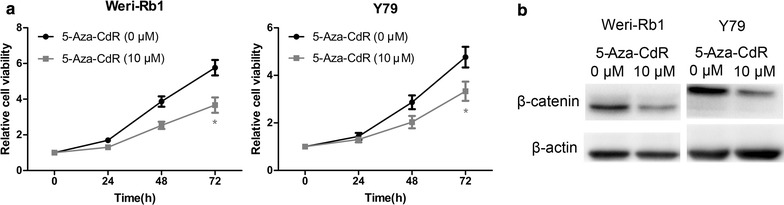
The effect of 5-Aza-CdR on cell proliferation and Wnt/β-catenin pathway in Weri-Rb1 and Y79 cells. **a** 5-Aza-CdR inhibited the cell proliferation. **b** 5-Aza-CdR depressed the activity of the Wnt/β-catenin pathway. *P < 0.05

In this section, we evaluated the effect of a DNA demethylating agent (5-Aza-CdR) on MEG3 expression at the cellular level. First, we found that MEG3 promoter was methylated in Weri-Rb1 and Y79 cells (Fig. 2a). Following treatment of Weri-Rb1 and Y79 cells with 5-Aza-CdR, the methylation level of MEG3 promoter was significantly decreased compared with control cells (Fig. 2a). Meanwhile, the expression level of MEG3 was significantly higher in 5-Aza-CdR treated cells than those in control cells (Fig. 3b).

### MEG3 methylation regulated proliferation and apoptosis in retinoblastoma cells

**Fig. 4:**
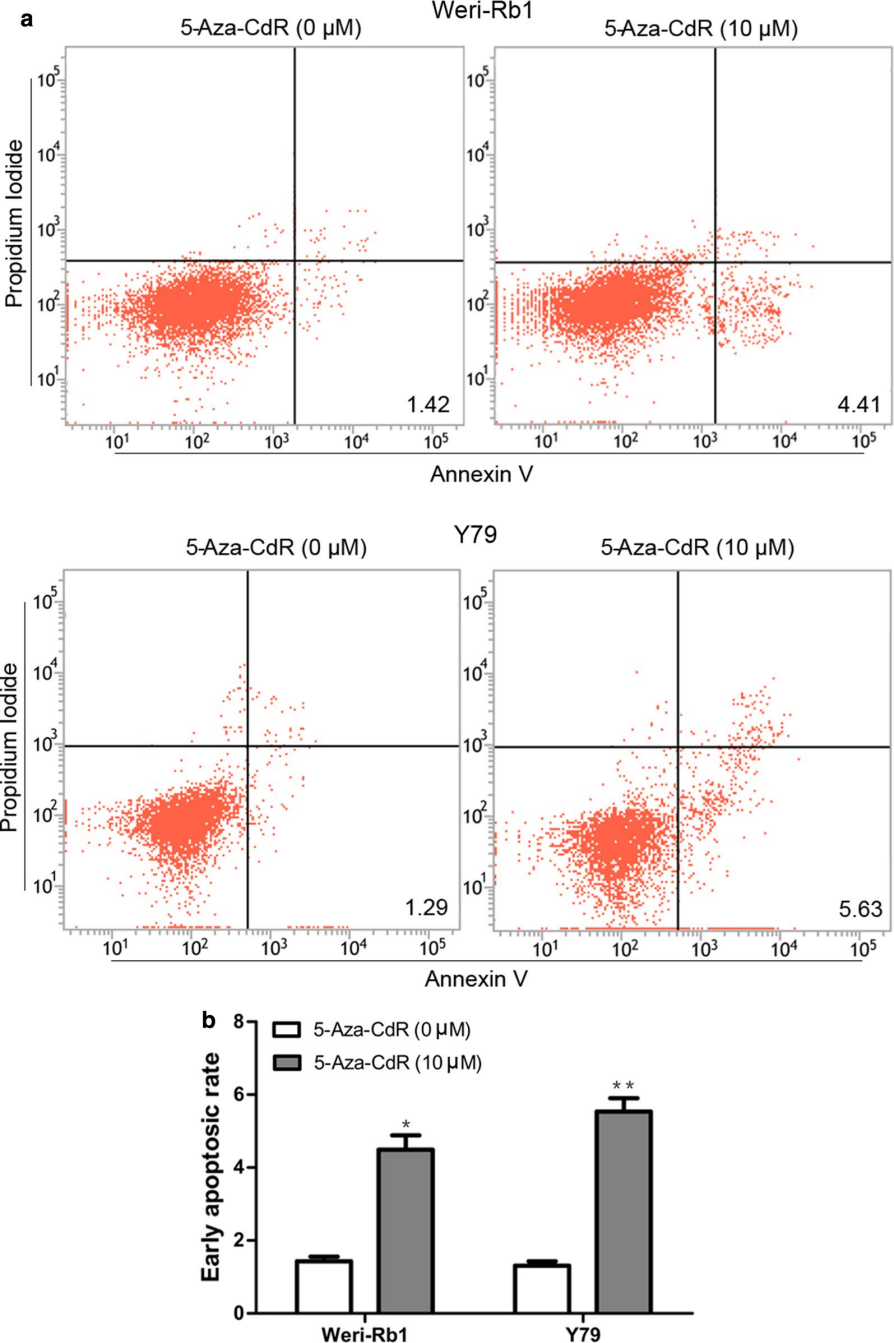
The effect of 5-aza-CdR on cell apoptosis in Weri-Rb1 and Y79 cells. **a** and **b** 5-Aza-CdR increased the number of early apoptotic cells of Weri-Rb1 and Y79 cells. *P < 0.05, **P < 0.01

**Fig. 5:**
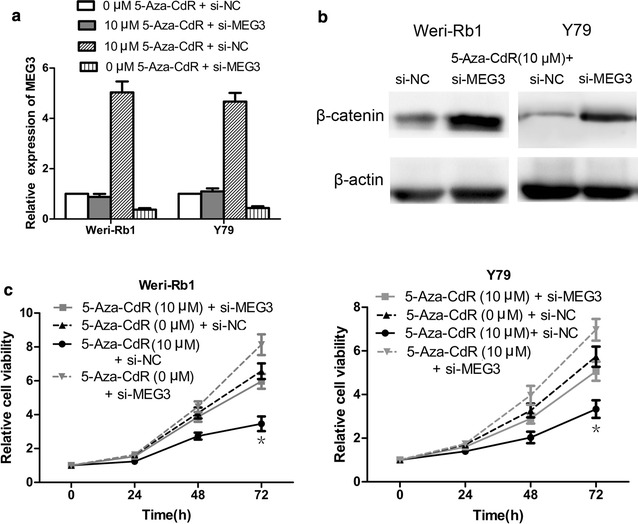
MEG3 re-expression played an important role in the antitumor effect of 5-Aza-CdR in Weri-Rb1 and Y79 cells. **a** Si-MEG3 was just offset the MEG3 re-expression effect of 5-Aza-CdR. **b** Si-MEG3 could block the effect of 5-Aza-CdR on β-catenin expression. **c** Si-MEG3 could reverse the effect of 5-Aza-CdR on cell proliferation. *P < 0.05

**Fig. 6:**
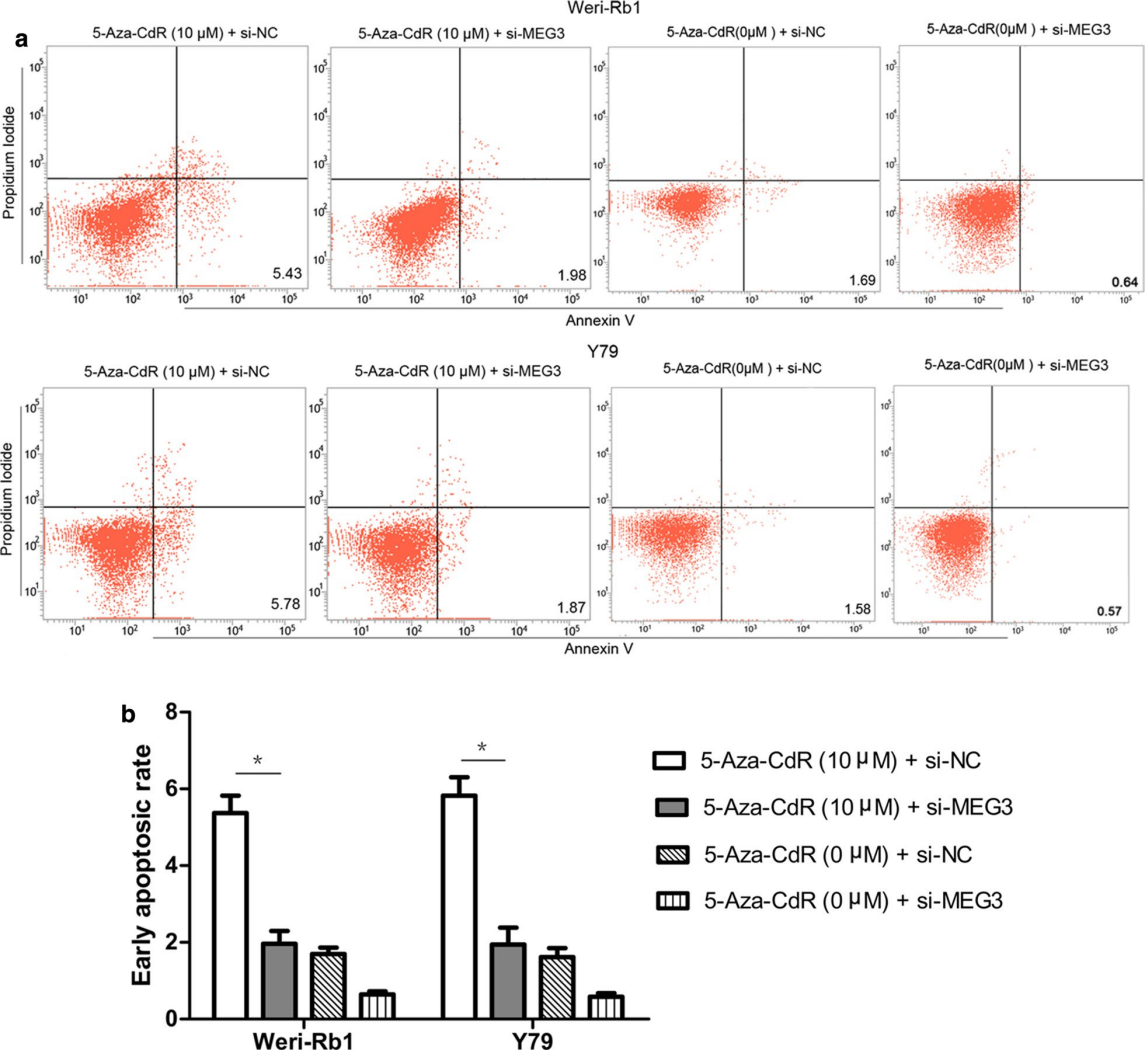
MEG3 re-expression contributed to inhibitory effect of 5-aza-CdR on apoptosis in Weri-Rb1 and Y79 cells. **a** and **b** Si-MEG3 could depress the effect of 5-Aza-CdR on cell apoptosis. *P < 0.05

First of all, CCK-8 assay and flow cytometric analysis in Weri-Rb1 and Y79 cells revealed that treatment with 5-Aza-CdR significantly reduced cell proliferation and increased the number of early apoptotic cells compared with control cells, respectively (Figs. 3a, 4a, b). Then Western blot analysis showed that the expression of β-catenin decreased obviously in cells treated with 5-Aza-CdR compared with control cells (Fig. 3b). In order to further reveal the role of MEG3 demethylation in the tumor suppression effect of 5-Aza-CdR, we designed the experiments in below. In initial step, qRT-PCR was used to confirm that si-MEG3 was just able to reverse the effect of 5-Aza-CdR on the re-expression of MEG3 (Fig. 5a). Moreover, CCK-8 assay and flow cytometric analysis demonstrated significant promotion of cell proliferation and inhibition of cell apoptosis in 5-Aza-CdR+ si-MEG3 group compared with 5-Aza-CdR+ si-NC group (Figs. 5c, 6a, b). Meanwhile, the expression of β-catenin was significantly up-regulated in 5-Aza-CdR+ si-MEG3 group as compared with 5-Aza-CdR+ si-NC group by Western blot (Fig. 5b).

## Discussion

In our previous study [10], we found that MEG3 was down-regulated in retinoblastoma and associated with IIRC stages, nodal or distant metastasis and prognosis. Furthermore, we proved that MEG3 was a tumor suppressor by negatively regulating the activity of Wnt/β-catenin pathway in retinoblastoma. However, the exact mechanics of MEG3 lost in retinoblastoma were still unknown.

Epigenetic gene silencing of tumor suppressor genes which association with promoter CpG island (CGI) hypermethylation is considered to be an early event during tumor development and one of the hallmarks of cancer [11–14]. The promoter region of MEG3 is rich in CGI and there are two differentially methylated regions (DMRs) located upstream of the MEG3 gene (IG-DMR and MEG3-DMR) which are imprinting control centers for the DLK1-MEG3 locus [15, 16]. Moreover, recent studies also showed that promoter methylation plays a major role in silencing of the MEG3 gene in pituitary tumor [17], meningiomas [18], cervical cancer [19, 20] and neuroblastoma cell lines [15].

Based on these, we proposed a hypothesis that methylation within the promoter region may be associated with loss of MEG3 expression in retinoblastoma as well. In the present study, MSP was used to evaluate the promoter methylation status of MEG3 in retinoblastoma tissues and corresponding non-tumor tissues. We first found that hypermethylation of MEG3 promoter occurred significantly more frequently in retinoblastoma tissues than in normal tissues. Furthermore, we testified the positive correlation between the hypermethylation of MEG3 promoter and the inactivation of MEG3 expression in retinoblastoma tissues. Collectively, these results indicate that aberrant MEG3 promoter hypermethylation maybe the major mechanism for MEG3 inactivation in retinoblastoma. These conclusions were similar with other studies and were also corroborate with our previous study [10, 15, 17, 18].

We noticed that a few of samples (6/42) denoted “Methylated pattern” but “high MEG3 expression”. That maybe other regulatory mechanism but not promoter methylation predominates in those samples. The regulation of gene expression is achieved through a complex and elaborate regulatory network. Gene expression can be regulated at pre-transcriptional level, transcription level and translation level. In the same way, the expression of MEG3 is regulated at different level. For example, EWSAT1 regulated MEG3 expression through association with HNRNPK in a transcriptional level [21]. MiR-141 regulated MEG3 expression through repression of EZH2 expression, a subunit of the polycomb repressor complex 2 (PRC2), in prostate cancer [22]. Therefore, methylation of promoter plays a major role in silencing of the MEG3 gene, but not the only way. Histone modifications, LncRNA, MicroRNA also play a regulatory role in the expression of MEG3 to a certain extent. Anyway, 36 of 42 case still support that methylation of promoter remains the primary regulatory mechanism of inactivation of MEG3.

Moreover, Chi square test implied that hypermethylation of MEG3 promoter was not only related to but also a risk factor for retinoblastoma. This result was coincident with our previous study and proved that there is good relativity between the methylation level of MEG3 promoter and expression level of MEG3 gene in retinoblastoma on the other hand. It also implied that aberrant hypermethylation of MEG3 promoter was involved in tumorigenesis of retinoblastoma. For MEG3 is a key factors for prognosis [10], we speculated that MEG3 methylation may be associated prognosis of retinoblastoma as well. As expected, there was a trend towards better outcomes in patients with lower methylation levels compared with patients with higher methylation levels. In our previous study [10], we found that MEG3 expression, IIRC stages, and nodal or distant metastasis were independent prognostic factors for recurrence-free survival in retinoblastoma patients. In consideration of the stability of DNA in plasma, we wonder if MEG3 methylation can take the place of MEG3 expression as an independent prognostic factor. And multivariate analysis proved that MEG3 methylation was also an independent prognostic factor. It means MEG3 methylation can be a candidate of plasma biomarker for prognosis and is valuable in detection of tumor recurrence. In summary, it is recommended that patients with retinoblastoma and MEG3 hypermethylation should receive aggressive intervention following initial curative treatment to achieve more favorable outcomes and MEG3 methylation maybe a promising plasma biomarker for retinoblastoma.

Next, we demonstrated the hypothesis at the cellular level. Consistent with the result in tissues, we confirmed that MEG3 promoter was hypermethylation in both Weri-Rb1 and Y79 cells, and methylation inhibitor 5-Aza-CdR could restrain the methylation of MEG3 promoter. To further examine the role of hypermethylation in down-regulation of MEG3 in retinoblastoma, we evaluated the effect of the 5-Aza-CdR on MEG3 expression. We noted that MEG3 expression was robustly increased by incubation with 5-Aza-CdR and the increment was positive relation to the demethylation levels of MEG3 promoter. These data strongly suggest that MEG3 expression can be modulated by alterations in promoter methylation and support an epigenetic mechanism of regulation of MEG3 expression in retinoblastoma.

To clarify the role of MEG3 methylation in regulation of proliferation and apoptosis in retinoblastoma cells, we used si-MEG3 to offset the effect of the 5-Aza-CdR on MEG3 re-expression and observed the change of biological characteristics of retinoblastoma cells. Interestingly, we found that the antitumor effects of 5-Aza-CdR were significantly repressed by si-MEG3 in retinoblastoma cells. These results indicate that the tumor suppression effect of 5-Aza-CdR in retinoblastoma was attributed in part to the MEG3 re-expression. On the other hand, it demonstrated that MEG3 re-expression which due to demethylation could inhibit the growth of retinoblastoma cells. What’s more, it also implied that the loss of MEG3 expression which was the result of hypermethylation in the MEG3 promoter could lead to malignant proliferation of retinoblastoma cells.

In our previous study [10], we found that MEG3 could inhibit the activity of the Wnt/β-catenin pathway in retinoblastoma. Thus, we wonder if the MEG3 methylation can regulated the activity of the Wnt/β-catenin pathway as well. Our result indicated that methylation levels of MEG3 promoter also could regulated the activity of the Wnt/β-catenin pathway.

As an important way of DNA modification, DNA methylation can affect gene expression. The change of DNA methylation pattern, especially the abnormal increase of methylation level of some tumor suppressor genes, plays an important role in the process of tumor development and progression. The reason for this change might be attributed to the increase of DNA methyltransferase activity. Therefore, inhibition of methyltransferase activity to restore the expression of tumor suppressor genes may interfere with the occurrence and development of tumors. Recently, the inhibitors of methyltransferase, such as 5-Aza-CdR, have attracted a lot of attention. 5-Aza-CdR has been shown to inhibit the proliferation of various tumor cells. In present study, we found that 5-Aza-CdR can regulate the cell growth in retinoblastoma. Then we revealed that 5-Aza-CdR maybe repress cell growth thought the pathway as follows: 5-Aza-CdR → demethylation of MEG3 promoter → up-regulation of MEG3 → inactivation of the Wnt/β-catenin pathway → growth inhibition of retinoblastoma cells. That means the methylation inhibitor of MEG3 promoter can be a novel therapeutic application for retinoblastoma.

Recent study showed that activation of Rb protein causes decreased expression of DNMT1, allowing for increased MEG3 expression and decreased cancer cell growth in lung cancer cell [23]. In view of the fact that deletion, mutation or inactivation of RB Gene is one of the important causes of retinoblastoma, the regulation between MEG3 and RB protein maybe involved in the development of retinoblastoma. It provides a new perspective in understanding the mechanism of MEG3 in retinoblastoma.

## Conclusions

In conclusion, our results revealed that aberrant promoter hypermethylation can lead to epigenetic inactivation of MEG3 and promotion of retinoblastoma cells growth. The evaluation of the MEG3 methylation status can be a useful biomarker in determining the prognosis of patients with retinoblastoma.

## Abbreviations

lncRNA: long non-coding RNA
MEG3: maternally expressed gene 3
qRT-PCR: quantitative real-time PCR
CCK-8: Cell Counting Kit-8
5-Aza-CdR: 5-aza-2-deoxy-cytidine
MSP: methylation specific PCR
IIRC: International Intraocular Retinoblastoma Classification
